# Thyroid and heart, a clinically relevant relationship

**DOI:** 10.1007/s40618-021-01590-9

**Published:** 2021-05-25

**Authors:** G. Corona, L. Croce, C. Sparano, L. Petrone, A. Sforza, M. Maggi, L. Chiovato, M. Rotondi

**Affiliations:** 1 Endocrinology Unit, Medical Department, Maggiore-Bellaria Hospital, Azienda-Usl Bologna, Bologna, Italy; 2 Unit of Internal Medicine and Endocrinology, Laboratory for Endocrine Disruptors, Istituti Clinici Scientifici Maugeri IRCCS, 27100 Pavia, Italy; 3 PHD Course in Experimental Medicine, University of Pavia, 27100 Pavia, Italy; 4 Department of Internal Medicine and Therapeutics, University of Pavia, Via S. Maugeri 4, 27100 Pavia, Italy; 5 Endocrinology Unit, Department of Experimental, Clinical and Biomedical Sciences, University of Florence, Florence, Italy

**Keywords:** Thyroid, Hypothyroidism, Hyperthyroidism, Cardiovascular risk, Coronary artery diseases

## Abstract

**Background:**

Thyroid disorders, both overt and subclinical, are highly prevalent conditions in the general population. Although a clear relationship between overt thyroid dysfunctions and cardiovascular complications has long been established, data regarding subclinical thyroid dysfunction are by far more controversial.

**Purpose:**

The present review will be aimed at providing a summary of most recent evidence coming from meta-analyses regarding the complex relationship between thyroid dysfunction and cardiovascular disease.

**Conclusions:**

The review will summarize, in the first part, the physiopathological link between thyroid hormone imbalances and the cardiovascular system. In the second part the review will outline the evidence coming from meta-analyses regarding the cardiovascular risk related with both overt and subclinical thyroid dysfunctions. Particular attention will be put towards studies showing data stratified for patient’s age, TSH levels and pre-existing cardiovascular disease. Finally, an overview regarding the effects of specific therapy for subclinical thyroid diseases in terms of amelioration of cardiovascular outcomes will be included.

## Introduction

Thyroid autoimmune diseases are highly prevalent clinical conditions, with an estimated prevalence of 9–25% in the adult female population [1, 2]. Lower rates were reported in the male population, consistently with the higher prevalence of autoimmune diseases described in females [1, 2]. Epidemiological data from both the United States [2, 3] and Europe [4] show that, in about half of the cases, these conditions, especially when at a subclinical stage, may remain undiagnosed and possibly represent contributing causes of several pathological conditions, including cardiovascular(CV) diseases (CVD).

Hypothyroidism represents the most frequent endocrine disease in the Western world, with a prevalence of about 4–5% in the general population and an annual incidence of 3.5/1.000 in women and 0.6/1,000 in males [3]. Hyperthyroidism is a fairly common endocrine disease with a prevalence of 0.5–1.5% and an incidence of 20/1,000,000/year in the general population with a male/female ratio of 1:5 [2, 4].

Thyroid hormones play a crucial role in glucose and lipid homeostasis and contribute to the regulation of heart function and the peripheral vascular system. The purpose of this article is to overview and systematically summarize the findings provided by the currently published meta-analyses addressing the role of thyroid dysfunctions on cardiac function and CV risk.

## Methods

A comprehensive narrative review was performed using Medline, Embase and Cochrane search and including the following words: ("thyroid gland"[MeSH Terms] OR ("thyroid"[All Fields] AND "gland"[All Fields]) OR "thyroid gland"[All Fields] OR "thyroid"[All Fields] OR "thyroid usp"[MeSH Terms] OR ("thyroid"[All Fields] AND "usp"[All Fields]) OR "thyroid usp"[All Fields] OR "thyroids"[All Fields] OR "thyroid s"[All Fields] OR "thyroidal"[All Fields] OR "thyroideal"[All Fields] OR "thyroidism"[All Fields] OR "thyroiditis"[MeSH Terms] OR "thyroiditis"[All Fields] OR "thyroiditides"[All Fields]) AND ("cardiovascular diseases"[MeSH Terms] OR ("cardiovascular"[All Fields] AND "diseases"[All Fields]) OR "cardiovascular diseases"[All Fields]). Publications from January 1, 1969 up to January 15^th^, 2021 were included. When available, meta-analytic data were preferred.

## Role of thyroid hormones on CV function

Thyroid hormones exert a direct action at the cardiac level through the combination of genomic and non-genomic effects, ultimately contributing to the regulation of cardiac function and CV hemodynamics [5]. Myocytes do not express deiodinase activity so the myocardial effect derives from the peripheral conversion of tetraiodothyronine (T4) into the active form of the hormone triiodothyronine (T3). At the cardiac level, stimulation of the β-type thyroid-hormone receptors determines an up-regulation of the calcium-dependent ATPase pumps of the endoplasmic reticulum and a reduced expression of the Phospholamban protein resulting in an inhibitory action. The result is an increased relaxation of the myocardium which, together with the increased expression of the α-isoforms of the heavy chains of myosin and with a faster contractile action, accounts for the positive inotropic effect of thyroid hormones at the myocardial level [1, 6]. The positive chronotropic effect derives mainly from a direct action of T3 on the genes that regulate the activity of the cardiac-pacemaker. Although a role of cathecolaminergic stimulation has been hypothesized, this seems unlikely. Indeed, T3 induces an increase in the expression of β1-adrenergic receptors on myocardiocytes, increasing myocardial sensitivity to the action of catecholamines, as it simultaneously inhibits the cardiac expression of the catalytic isoforms of adenylate–cyclase, keeping the endocellular response unchanged [1, 6]. At the peripheral level, the stimulation of the α-1 thyroid receptors, expressed by the endothelial and smooth muscle cells of the vessels, reduces peripheral resistance causing a decrease in diastolic pressure [1, 6]. At the renal level, this effect leads to a reduced perfusion with consequent activation of the renin–angiotensin–aldosterone system (to which a direct effect of T3 via thyroid β-1 receptors probably also contributes) with a sodium-retentive action and an increase in circulating volume [1, 6].

Another possible link between thyroid function and the cardiovascular system is represented by the regulation of the circadian rhythm. Indeed, recent papers have highlighted the importance of the interconnection between thyroid function and circadian clocks, showing the occurrence of circadian disruption caused by thyroid dysfunction [7]. Chronic circadian disruption has been clearly proven to be related with an increased risk of obesity, diabetes mellitus and cardiovascular diseases, providing further support to the link between thyroid dysfunction and cardiovascular risk [8].

## Thyroid alterations and CVD

### Hypothyroidism

As previously reported, the presence of hypothyroidism can impair cardiac contractility and reduce myocardial relaxation. Echocardiographic studies have shown that overt hypothyroidism is often associated with early diastolic dysfunction which, together with the frequent occurrence of diastolic hypertension and/or other risk factors in these patients, can lead to difficult-to-treat heart failure [1, 3]. Overt hypothyroidism causes an interstitial accumulation of glycosaminoglycans that attracts water from the endovascular compartment, reducing the effectiveness of medical therapy. In the most severe forms, chronic and pericardial interstitial edema, associated with reduced myocardial contractility, can progress to cardiac tamponade [1, 3]. The presence of hypothyroidism also involves a reduction in the activity of the myocardial pacemaker and a lengthening of the QT intervals which can lead to the appearance of advanced atrio-ventricular blocks or torsades-de-pointes resulting in ventricular tachycardia [1, 3]. Finally, the presence of hypothyroidism negatively impacts glucose and lipid metabolism possibly favoring the onset of insulin resistance and hypercholesterolemia which may, at least in part, be reverted by replacement therapy [9, 10].

In line with the above stated concepts, a recent meta-analysis, including 55 studies and a total of 1,898,314 subjects, concluded that the presence of overt hypothyroidism is associated with an increased risk of myocardial ischemia (13%), myocardial infarction (15%), arrhythmias (96%) and overall mortality (25%) when compared to euthyroidism [11].

### Hyperthyroidism

Patients with overt hyperthyroidism, at a rate ranging from 10 to 25%, experience atrial fibrillation (AF) with even higher percentages in males over 60 years of age and in those with higher free T4 (FT4) values. Conversely, only 5% of subjects with thyrotoxicosis under the age of 60 have AF [1, 6]. Furthermore, it should be remembered that thyrotoxicosis determines an imbalance of coagulative homeostasis towards a state of hypercoagulability and reduced fibrinolysis characterized by an increase in the circulating levels of factor VIII, IX, fibrinogen and Von Willebrand factor [1, 6], further complicating the clinical picture. The presence of tachyarrhythmia associated with the previously described hemodynamic alterations is often the cause of high-rate heart failure, especially in patients with concomitant risk factors [1, 6].

A meta-analysis involving 7 studies and 31,138 subjects actually concluded that the presence of overt hyperthyroidism increases overall mortality by 13% and the mortality for CV causes by 21% [12]. Another more recent meta-analysis including 37 studies and enrolling 113,393 hyperthyroid subjects showed that overt hyperthyroidism increases the risk of ischemic heart disease, stroke, and cardiovascular mortality [13].

### Forms of subclinical thyroid alterations

The relationship between overt hyperthyroidism and hypothyroidism and CV pathology is well established and briefly summarized in the previous paragraphs. By contrast, the relationship between subclinical changes in thyroid function and CV risk is far more controversial. This aspect will be analyzed in detail in the following sections.

#### Subclinical hypothyroidism (SC-HT)

Subclinical hypothyroidism (SC-HT) is a condition biochemically characterized by an increase in thyroid-stimulating-hormone (TSH) levels with free hormones within the normal range [14]. Based on TSH levels it is possible to distinguish mild forms (TSH 4–10 mU/L) and more severe forms with TSH > 10 mU/L [15]. The estimated prevalence is about 5–10% of the general population with a clear female gender prevalence. The risk of progression from subclinical to overt hypothyroidism is around 1–5% per year, showing a positive association with TSH levels, the presence of thyroid antibodies, positivity at high titers and a diffuse hypoechoic pattern of the thyroid at ultrasound [15, 16].

Since 2006, 11 meta-analyses have been published on the association between CV risk and subclinical hypothyroidism (Table 1; [11, 17–25]). The number of studies considered varied from 4 to 37 and the number of subjects included from 2,116 to 1,473,648 (Table 1). Seven meta-analyses [11, 17, 19–22, 24, 26] report data on composite coronary events, two on the risk of AF [24, 27] and two on the risk of heart failure (SCC) [23, 24] (Table 1). In addition, five ([19–22, 24]) and eight [11, 18, 19, 21, 22, 24, 25] meta-analyses, respectively, describe the possible relationship between SC-HT and CV and overall mortality risk (Table 1). Finally, one meta-analysis reports data on the risk of a composite outcome of fatal and non-fatal CV events (MACE) [25].

**Table 1:** Characteristics of available meta-analyses on the relationship between subclinical hypothyroidism and cardiovascular (CV) morbidity and mortality. MACE = major cardiovascular events

Inclusion criteria	Rodondi et al. [17] (11)	Haentjens et al. [18] (12)	Ochs et al. [19] (13)	Razvi et al. [5] (14)	Singh et al. [21] (15)	Rodondi et al. [22] (16)	Gencer et al. [23] (17)	Ning etal. [11] (9)	Sun et al. [24] (18)	Moon et al. [25] (19)	Du Puy et al. [28] (25)
**N. of included studie**s	14	9	12	15	6	11	6	37	16	35	4
N. of analyzed patients	13,011	14,912	14,449	29,022	14,386	55,287	25,390	1,473,648	71,808	555,530	2,116
Age mean/range (years)	18–95	–	18–95	60	17–89	19–94	21–100	45–83	45.4–85	41–83.4	80–109
Follow Up mean/range (years)	–	2–20	2–20	4–20	4–20	2.5–20	2–16.3	2.5–20	3.2–20	–	5
TSH cut off mU/L Subclinical hypothyroidism	2.8–6.0	4–5.10	4–6	3.7–8.9	4–5	0.50	4.5–19.9	3.1–6.0	4–6	–	4.8

	***Yes***	*No*	***Yes***	*No*	***Yes***	*No*	***Yes***	*No*	***Yes***	*No*	***Yes***	*No*	***Yes***	*No*	***Yes***	*No*	***Yes***	*No*	***Yes***	*No*	***Yes***	*No*
Unadjusted risk	***X***		***X***		***X***			***X***		***X***	***X***			***X***	***X***		***X***			***X***		***X***
Adjusted risk	***X***			***X***	***X***		***X***		***X***		***X***		***X***			***X***	***X***		***X***		***X***	
Age stratified risk		***X***		***X***	***X***		***X***			***X***	***X***		***X***		***X***		***X***		***X***		***X***	
TSH stratified risk		***X***		***X***	***X***			***X***		***X***	***X***		***X***			***X***		***X***		***X***		***X***
**CV events analyzed**																						
MACE		***X***		***X***		***X***		***X***		***X***		***X***		***X***	***X***			***X***	***X***			***X***
Coronary events	***X***			***X***	***X***		***X***		***X***		***X***			***X***	***X***		***X***			***X***		***X***
Atrial fibrillation		***X***		***X***		***X***		***X***		***X***		***X***		***X***		***X***	***X***			***X***		***X***
Heart failure		***X***		***X***		***X***		***X***		***X***		***X***	***X***			***X***	***X***			***X***		***X***
CV mortality		***X***		***X***	***X***			***X***	***X***		***X***			***X***	***X***		***X***			***X***		***X***
Overall mortality		***X***	***X***		***X***		***X***		***X***		***X***			***X***	***X***		***X***		***X***		***X***	

**Fig. 1:**
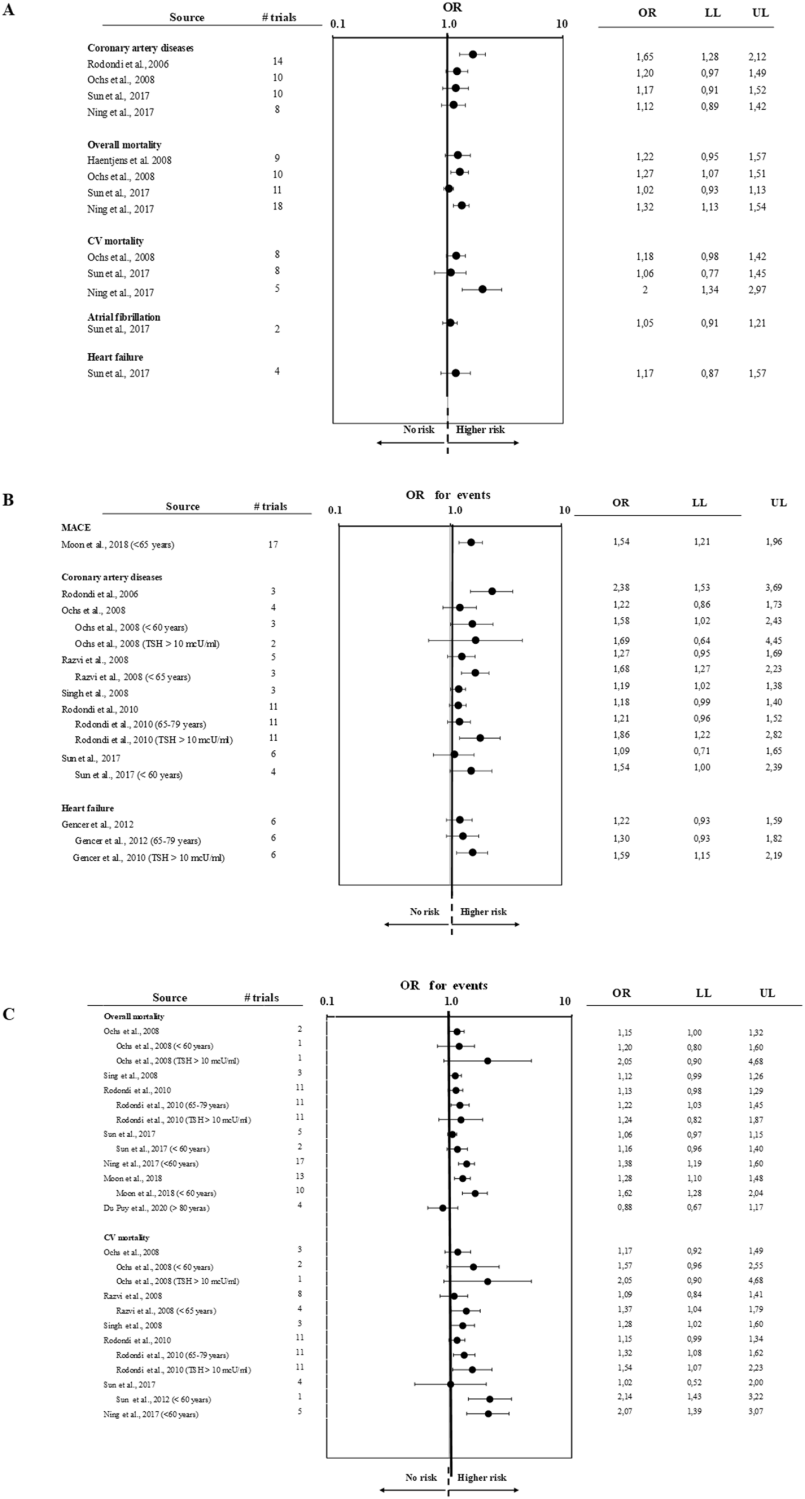
Forest plot of estimated unadjusted (A) and multiple adjusted (B–C) odds ratios (95% confidence intervals) for several cardiovascular (CV) outcomes according to presence of subclinical hypothyroidism as derived from available meta-analyses. MACE = major adverse cardiovascular events. LL = lower limits; UP = upper limits

Most studies reported a relationship between SC-HT and increased risk of coronary events and CV mortality (Fig. 1, Panels A–C). This association was also confirmed by the only study reporting the association between SH and the most validated cardiovascular outcome (MACE; Fig. 1, Panel B). The risk seems particularly evident in younger subjects and when TSH values > 10 mU/L are considered (Fig. 1, Panel B). The presence of severe SC-HT (i.e., as defined by circulating TSH levels > 10 mU/L) would also be associated with an increased risk of congestive heart failure without a relationship between SC-HT and AF (Fig. 1, Panel B). The relationship between SC-HT and overall mortality is more conflicting although the two meta-analyses [11, 25] which considered a larger number of studies suggest an increased risk in younger subjects (Fig. 1, Panels A and C). Age, therefore, seems to represent a crucial factor in the relationship between CV risk and SC-HT. The reasons have not yet been fully clarified but two main hypotheses can be put forth. On the one hand, it is possible to hypothesize that SC-HT may cause more accelerated vascular, endothelial and myocardial damage in younger subjects. In addition, a potential contribution of the autoimmune process per se has been suggested due to a possible association between autoimmune thyroid disease and CV morbidity on the basis of coronary inflammatory cross-reactivity [1, 3].

On the other hand, the progressive increase in the number of conventional CV risk factors, which generally occurs with increasing age, can reduce the independent contribution of SC-HT to the stratification of CV risk [1, 3]. The mechanisms through which SC-HT can contribute to the increase in CV mortality and morbidity are several and, at least in part, similar to those reported for overt hypothyroidism.

Overall, it is interesting to underline that the association between SC-HT and increased risk for CV morbidity and mortality appears to be rather consistently reported, particularly by those meta-analyses involving a larger number of studies, even after correction for conventional CV risk factors.

Furthermore, the association between SC-HT and increased risk of MACE was found to be significant only for younger subjects (< 65 years) with a higher CV risk, unlike older individuals (> 65 years) (Fig. 1, Panel B) [25]. Similarly, no association between overall mortality and SC-HT was confirmed by an extremely recent meta-analysis performed on four prospective cohort studies including subjects older than 80 years, which showed no correlation between SC-HT and all-cause mortality[28].

Another very recent meta-analysis evaluated a similar issue but from a slightly different perspective: the authors aimed to evaluate the role of hypothyroidism as a risk factor for all-risk mortality and MACE in a population of patients suffering from ischemic heart disease. The authors included 7 studies in their analysis, showing that hypothyroidism (both subclinical and overt) was associated with a higher risk of all-cause mortality (HR = 1.47; 95% CI = 1.10–1.97, p = 0.009) and MACE (HR = 1.53, CI = 1.19–1.97; p < 0.001). However, the 3 studies separately analyzing the impact of overt versus SC-HT reported no significant differences for overall mortality or MACE between euthyroid and subclinical hypothyroid patients [29].

Similar results were reported by another recent meta-analysis evaluating if subclinical thyroid dysfunction could be a predictor of worse outcomes in patients with heart failure. The meta-analysis, including 21 studies and 46,302 patients, showed that SC-HT was associated with a significantly increased risk of all-cause mortality and cardiovascular events compared to euthyroid patients in this specific, high-risk population [30].

Data on general mortality need further studies to fully clarify the etiology of this correlation. The available evidence is insufficient to fully clarify the main causes of the observed increase in death risk related to SC-HT.

Despite the above reported significant clinical association, the therapeutic benefit, in terms of reduction in CV risk, of levothyroxine (LT4) therapy in patients with SC-HT remains controversial. No randomized controlled trials (RCTs) with CV mortality and morbidity after LT4 therapy as the primary endpoint have been published yet, but indirect evidence can be drawn through meta-analysis. A meta-analysis related to a limited number of RCTs has shown how LT4 therapy, when compared to placebo, in subjects with SC-HT, could be associated with an improvement in lipid, glycaemia and some echocardiographic parameters, such as myocardial relaxation and diastolic function [31]. Another recent meta-analysis, including 166 studies (23 randomized and 143 non-randomized), with a total of 12,855 patients enrolled, confirmed that treatment of both overt and subclinical hypothyroidism with LT4 caused a significant reduction in serum lipids (including total cholesterol, Low Density Lipoproteins or LDL and triglycerides), with a similar but smaller effect in subclinical hypothyroidism when compared with overt hypothyroidism [32].

Furthermore, prospective population registries seem to suggest a beneficial role of LT4 therapy on CV morbidity and mortality in subjects with SC-HT. An analysis of the United Kingdom general practitioner registry showed that LT4 therapy is associated with a lower number of coronary ischemic events in young subjects with SC-HT (40–70 years) but not in older ones [33].

A very recent meta-analysis by Peng et al. evaluated the impact of thyroid hormone therapy on mortality in adults with SC-HT. Five observational studies and two randomized controlled trials (with a total of 21,055 patients) were included. The results showed that LT4 therapy was associated with a significant reduction in all-cause (RR = 0.50, CI 0.29–0.85, p = 0.011) and cardiovascular (RR = 0.54, 95% CI 0.37–0.80, p = 0.002) mortality in patients younger than 65 years, but not in older patients [34].

Based on currently available data, the guidelines of the European Thyroid Association (ETA) suggest starting treatment for SC-HT in young patients (< 65 years) with higher TSH (> 10 mU/L) even in the absence of specific symptoms. Treatment for milder forms of SC-HT should be reserved for symptomatic younger subjects (< 65 years). Older subjects (> 80 years) with a TSH ≤ 10 mU/L should be closely monitored and generally not treated. In the presence of an underlying CVD, especially in elderly patients, both the ETA and the American Thyroid Association (ATA) guidelines agree with the “start-low, go-slow” recommendation, suggesting a starting dose of 12.5–25 μg/day [2, 15]. LT4 should be progressively increased by 12.5–25 μg daily every 4–8 weeks and TSH should be targeted to 4–6 mU/l in persons older than 70–80 years.

#### Subclinical hyperthyroidism (SC-HPT)

Subclinical hyperthyroidism (SC-HPT) is a condition biochemically defined by a reduction in TSH levels with free fractions of thyroid hormones that remain within the normal range. On the basis of TSH levels, it is possible to distinguish grade 1 forms with measurable, although reduced, TSH levels (TSH 0.1–0.45 mU/L), and grade 2 forms with suppressed TSH (< 0.1 mU/L) [2]. The prevalence varies between 0.6 and 2% of the general population with an annual risk of progression towards frank forms of thyrotoxicosis of 0.5–7% depending on several factors, which include the degree of iodine intake, TSH levels and the presence of antibody positivity [2].

**Fig. 2:**
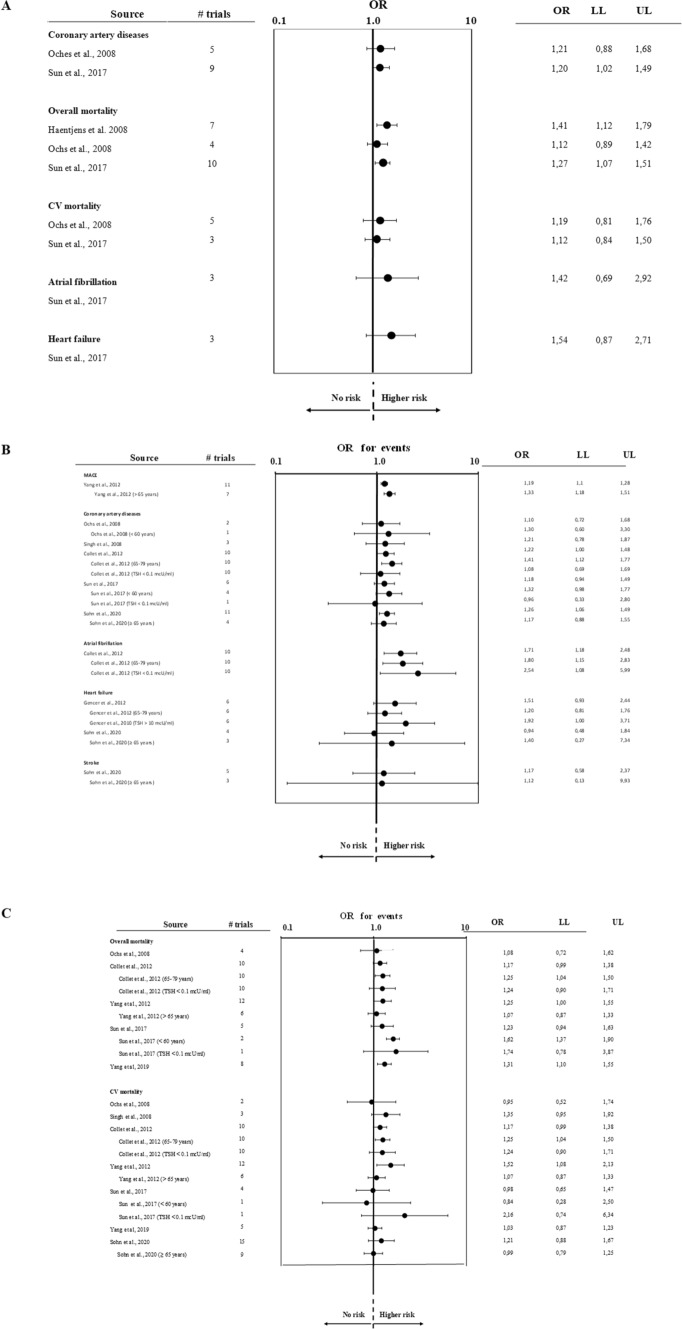
Forest plot of estimated unadjusted (A) and multiple adjusted (B–C) odds ratios (95% confidence intervals) for several cardiovascular (CV) outcomes according to presence of subclinical hyperthyroidism as derived from available meta-analyses. MACE = major adverse cardiovascular events. LL = lower limits; UP = upper limits

Since 2006, 9 meta-analyses on the association between CV morbidity and mortality and SC-HPT were published (Table 2; [18–21, 23, 24, 26, 35]). The number of studies considered varied from 4 to 17 and that the number of subjects included from 2,116 to 71,808 (Table 2). Five of them [13, 19, 21, 24, 26] report data on composite coronary events, two on the risk of AF [24, 26] and three on congestive heart failure [24, 35] (Table 2). In six meta-analyses the possible relationship between SC-HPT and CV mortality [19, 21, 24, 26, 35] and overall mortality risk [18, 19, 24, 26, 35] (Table 2) was investigated. Finally, a single meta-analysis reports data on MACE [35] or stroke [13] (Table 2). As might be expected, there is a close association between SC-HPT and risk of AF and congestive heart failure, especially in Grade 2 forms (Fig. 2, Panels A and B). Furthermore, most of the available meta-analyses confirm an increased risk of CV mortality and morbidity in SC-HPT (Fig. 2, Panels A and C). Finally, no relationship between SC-HPT and stroke was observed (Fig. 2, Panel B).

**Table 2:** Characteristics of available meta-analyses on the relationship between SC-HPT and cardiovascular (CV) morbidity and mortality. MACE = major cardiovascular events

Inclusion criteria	Haentjens et al. [18] (12)	Ochs et al. [19] (13)	Singh et al. [21] (15)	Collet et al. [26] (28)	Gencer et al. [23] (17)	Yang et al. [35] (29)	Sun et al. [24] (18)	Du Puy et al. [28] (25)	Sonh et al. [13] (10)
**N. of included studies**	9	12	6	10	6	17	16	4	37
N. of analyzed patients	14,912	14,449	14,386	52,674	25,390	52,808	71,808	2,116	1,626,005
Age mean/range (years)	–	18–95	17–89	59	21–100	49–85	45.4–85	80–109	51.3–85.5
Follow Up mean/range (years)	2–20	2–20	4–20	8.8	2–16.3	2–20	3.2–20	5	–
TSH cut off mU/L Subclinical hyperthyroidism	0.3–0.5	0.3–0.5	4–5	0.45	0.45	0.1–0.5	0.25–0.5	0.3	–

	Yes	No	Yes	No	Yes	No	Yes	No	Yes	No	Yes	No	Yes	No	Yes	No	Yes	No
Unadjusted risk	***X***		***X***			***X***		***X***		***X***		***X***	***X***			***X***		***X***
Adjusted risk		***X***	***X***		***X***		***X***		***X***		***X***		***X***		***X***		***X***	
Age stratified risk		***X***	***X***			***X***	***X***		***X***		***X***		***X***		***X***		***X***	
TSH stratified risk		***X***		***X***		***X***	***X***		***X***		***X***		***X***		***X***			***X***
**CV events analyzed**																		
MACE		***X***		***X***		***X***		***X***		***X***	***X***					***X***		***X***
Coronary events		***X***	***X***		***X***		***X***			***X***		***X***	***X***			***X***	***X***	
Atrial fibrillation		***X***		***X***		***X***	***X***			***X***		***X***	***X***			***X***		***X***
Heart failure		***X***		***X***		***X***		***X***	***X***			***X***	***X***			***X***	***X***	
CV mortality		***X***	***X***		***X***		***X***			***X***	***X***		***X***			***X***	***X***	
Total mortality	***X***		***X***		***X***		***X***			***X***	***X***		***X***		***X***			***X***

Overall, unlike what has been observed in SC-HT, the CV risk appears to be greater in older rather than younger subjects and it would likely be attenuated by the adjustment for confounding risk factors (Fig. 2, Panels B and C). These would support the notion that SC-HPT could act as a triggering event in already compromised subjects with multiple risk factors.

Few intervention studies with CV outcomes addressed the issue of the therapeutic benefit of restoring euthyroidism in terms of reduction of the CV risk in patients with SC-HPT [36]. In 2004, a consensus of experts proposed the need for anti-thyroid drug use in elderly subjects with grade 2 forms [37]. These considerations were essentially based on the possibility that treatment of these subjects would reduce the risk of AF and osteoporosis. The evidence of the last 15 years has essentially confirmed this hypothesis. Thus, both the European and American guidelines suggest early treatment of an SC-HPT, especially in the elderly (≥ 65 years) with TSH < 0.1 mU/l and with concomitant CV risk factors. Similarly, treatment is recommended in younger subjects with high CV risk even for milder forms of SC-HPT (grade 1). Conversely, in younger subjects and in the absence of CV risk factors, strict surveillance without immediate treatment can be considered [2, 38]. However, Biondi et al. suggested that treatment of subclinical hyperthyroidism should be considered also in young and middle-aged patients to prevent the cardiac consequences of prolonged SC-HPT [39].

## Conclusions

Overt hypo- and hyperthyroidism significantly increase the risk of CV mortality and morbidity by both direct (myocardial and coronary effects) and indirect (influence on the peripheral vascular system, lipid and glucose metabolism and coagulation homeostasis) mechanisms. More controversial is the role of subclinical thyroid dysfunctions. At present, the age of the patient appears as a major determinant in clinical decision-making. Indeed, currently available data demonstrate how treating SC-HT can be of greater benefit, especially in younger subjects with lower CV risk. Conversely, the treatment of SC-HPT guarantees greater advantages in the elderly patient especially when comorbidities are present.
